# The unbearable emptiness of tweeting—About journal articles

**DOI:** 10.1371/journal.pone.0183551

**Published:** 2017-08-24

**Authors:** Nicolas Robinson-Garcia, Rodrigo Costas, Kimberley Isett, Julia Melkers, Diana Hicks

**Affiliations:** 1 INGENIO (CSIC-UPV), Universitat Politècnica de València, Valencia, Spain; 2 Centre for Science and Technology Studies (CWTS), Leiden University, Leiden, The Netherlands; 3 School of Public Policy, Georgia Institute of Technology, Atlanta, Georgia, United States; KU Leuven, BELGIUM

## Abstract

Enthusiasm for using Twitter as a source of data in the social sciences extends to measuring the impact of research with Twitter data being a key component in the new altmetrics approach. In this paper, we examine tweets containing links to research articles in the field of dentistry to assess the extent to which tweeting about scientific papers signifies engagement with, attention to, or consumption of scientific literature. The main goal is to better comprehend the role Twitter plays in scholarly communication and the potential value of tweet counts as traces of broader engagement with scientific literature. In particular, the pattern of tweeting to the top ten most tweeted scientific dental articles and of tweeting by accounts is examined. The ideal that tweeting about scholarly articles represents curating and informing about state-of-the-art appears not to be realized in practice. We see much presumably human tweeting almost entirely mechanical and devoid of original thought, no evidence of conversation, tweets generated by monomania, duplicate tweeting from many accounts under centralized professional management and tweets generated by bots. Some accounts exemplify the ideal, but they represent less than 10% of tweets. Therefore, any conclusions drawn from twitter data is swamped by the mechanical nature of the bulk of tweeting behavior. In light of these results, we discuss the compatibility of Twitter with the research enterprise as well as some of the financial incentives behind these patterns.

## Introduction

Twitter, though not the most successful social media company [1], has seen unique success in enabling several public movements—the Arab spring, the Spanish 15M, Black Lives Matter and various mass expressions of consumer anger over corporate missteps. Such high visibility social media activity over the past decade combined with younger generations’ social media uptake has left more senior professionals worried about being left behind. The professional literature has therefore seen a steady stream of exhortations to join social media accompanied by suggested professional use cases.

The health professions have shown a genuine interest in the use of information technology to improve health care [2]. So it was not surprising to find doctors exhorting other doctors to join Twitter [3]. While voicing concerns with maintaining privacy and professionalism on social media, proponents described largely idyllic scenarios emphasizing Twitter’s potential for aiding career development [4], connecting with colleagues, building relationships with patients [5], enabling virtual journal clubs and scientific conferences [6], complementing traditional teaching methods [7,8], critically appraising and reviewing research [9], or all of the above [6]. Some have argued that Twitter promised to speed knowledge transfer and bridge the communication gap between researchers and stakeholders [10] leading others to suggest strategies and tips to improve the dissemination of research findings [11], and to engage readers to follow journals’ Twitter accounts [12].

This paper focuses narrowly on one suggested use of Twitter—tweeting about journal articles. Twitter being touted as “a great source for both disseminating and discovering information” [13] where “one can share information on research and papers from peers and colleagues that is interesting or cutting-edge” [14]. Across many research communities Twitter has been seen as a promising channel for increasing visibility and reaching broader audiences interested in discussing scientific literature [15,16]. Twitter is one of the most predominant sources of *altmetric* indicators, that is, mentions in social media platforms because it contains the most mentions of scientific papers [17,18]. The best covered literature in Twitter is biomedical [19,20] and social science [21]. Therefore, Twitter provides a key component of social media data that analysts hope to use to monitor the spread of scientific information outside academia.

This paper critically examines tweets containing links to research articles in the field of dentistry to assess the extent to which tweeting about scientific papers signifies engagement with, attention to, or consumption of scientific literature. The main goal is to better assess the proposal that counts of tweets trace engagement of broader audiences with scientific literature [22].

## Background

The argument that tweeting about papers is a good idea led naturally to the suggestion of counting those tweets and using the result as indicators of impact outside the academy [23,24]. The Altmetric Manifesto [25] promoted the importance of social media for accelerating the assessment of journal articles. Immediacy tempted many to see Twitter mentions of scholarly papers as traces of fast-moving conversations about scientific literature:

Twitter citations are also uniquely conversational, reflecting a broader discussion crossing traditional disciplinary boundaries. Twitter citations are much faster than traditional citations, with 40% occurring within one week of the cited resource’s publication. Finally, while Twitter citations are different from traditional citations, our participants suggest that they still represent and transmit scholarly impact… offering faster, broader, and more nuanced metrics of scholarly communication to supplement traditional citation analysis. For example, up-to-date metrics including Twitter citations might augment a tenure or promotion portfolio.

(Priem & Costello, 2010, p. 4)

In turn, this advocacy fed back to the professions. The work by Priem and Costello [26] has been a resource for doctors recommending Twitter use [11,27].

The vision seemed rapidly to become reality with the creation of Impactstory.org (formerly TotalImpact), a nonprofit corporation aimed at ‘helping to build a new scholarly reward system that values and encourages web-native scholarship’ (https://www.impactstory.org/about). In parallel, Altmetric.com was founded to provide data concerning mentions of research articles in social and news media [18]. Providers and advocates of altmetrics have admitted that there ‘has been some confusion over what is being claimed’ [28]. Still, their commercial interest dictated marketing efforts directed at convincing librarians of the value of social media and the virtues of integrating altmetrics into assessment of scholarship [29]. Librarians have become an important ally in the promotion of altmetrics, and a gap has opened between what librarians recommend researchers use and what researchers actually use [30]. Twitter and social media in general are now perceived as promising sources of evidence for assessing research [31].

However, early hopes that tweets would be faster versions of citations and so would predict citation counts have been dashed. Much research has examined the correlation between tweet and citation counts; a meta-analysis of this work found the correlation to be negligible [17,32]. Haustein and colleagues [33] stated that “the high number of tweets did not seem to be caused by their intellectual contribution or scientific quality” and the diversity of motivations for tweeting a paper made the value of tweeting papers inconclusive.

The lack of knowledge about who tweeted scientific literature [34] or the purposes that motivated them to do so, undermined the potential value of counts of tweets [35]. Tweets may have originated from publishers or corporations promoting their content, or from bots, both of which compromised any claim that tweeting of papers indicates attention or impact [36]. Recognition of the heterogeneity, volatility and inconsistency of tweets created calls for a more secondary role complementing traditional metrics rather than becoming a true alternative [37]. Researchers studying the use of altmetrics for research evaluation purposes have shown great skepticism as to their reliability and consistency when used to inform science policy makers and evaluators[19,38,39]. The growth in studies using twitter data in research evaluation as well as methodological cautions mirror broader trends in use of social media as a source of social science data [40,41].

## Data and methods

This work was part of a larger project addressing dissemination channels and clinical information sources in dentistry in the United States. In 2016, Web of Science included 84 journals in its category Dentistry, Oral Surgery & Medicine. In addition, PubMed indexed 47 journals that were active and were published in the United States. The complete list of journals can be found in the supplemental material [42]. References to papers in these journals were downloaded from Web of Science and PubMed. Both databases provide PubMed IDs and these were used to find tweets about the papers in the Altmetric.com database housed at CWTS, Leiden University that indexes the full text of tweets that contain a URL linking to a paper. This data was collected in compliance with the terms of service of Altmetric.com. Altmetric.com is one of the main providers of altmetric data. It tracks mentions and links to scientific literature from a selection of social media platforms, news media coverage and policy documents. Twitter is the predominant source of social media data in Altmetric.com [18]. Altmetric.com uses the Twitter API to collect tweets, retweets and quoted tweets that contain a direct link to a scholarly article from a publisher that they track [43]. Altmetric.com tracks URLs contained in the tweet as well as unique identifiers such as PubMed IDs, DOIs or ArXiv IDs.

For each tweet, the text tweeted, account name, and time posted were obtained. The data also flagged retweets. To this was added the cited journal article’s title and bibliographic reference. Because the larger project is examining the US dental profession, this Twitter data was restricted to accounts originating in the US. The final dataset contained 2,202 US based accounts that sent 8,206 tweets about 4,358 dental papers between June 2011 and June 2016. We refer the reader to the data set analyzed [42].

In what follows we discuss tweeting about dental papers based on a reading of the tweets, on looking at the spacing of tweets over time, and on examining the characteristics of the accounts posting the tweets. The analysis is organized into two sections. The first explores tweets about the top 10 most tweeted papers. The second explores how accounts engage with the content of tweeted papers, in particular how mechanical or human was the content of an account’s tweets.

## Results—Top 10 most tweeted papers

First the pattern of tweeting that brought the top 10 most tweeted scientific dental papers to prominence is explored. There were three patterns: single issue tweeters, professional social media account management and broader tweeting, though with little original content. None of the patterns evidenced engagement with the journal article. What follows describes each of these three patterns.

### Single issue campaigners

Monomania seemed to underpin high tweet counts. The most tweeted paper in this set was:

*Aminoshariae, A. and Khan, A., 2015. Acetaminophen: old drug, new issues. Journal of Endodontics, 41(5), pp.588-593*.

The paper was tweeted 264 times by US based tweeters. The paper’s altmetric score was 25: “In the top 5% of all research outputs scored by Altmetric” (https://www.altmetric.com/details/3753910/Twitter). However, a closer look raised doubts. 193 (73%) of the tweets came from one account, @autismepi who included a link to this paper 65 times in tweets that read: “Paracetamol research: [URL]” and *33* times in tweets that read: “#Acetaminophen- ‘may not be considered a safe drug in #pregnancy’-offspring behavioral disorders, hormone disruption [URL]”.

A second less extreme account, @jen_in_TX, tweeted this paper 58 times. The accounts retweeted each other sometimes. The account profiles convey the content of the tweets about this paper: Tylenol may not be considered a safe drug during pregnancy, which was an accurate conclusion to draw from this paper. Discarding all but one of the tweets from these two accounts, the paper would be left with 15 tweets.

The same phenomenon was visible in the second most tweeted paper:

*Hujoel, P., 2009. Dietary carbohydrates and dental-systemic diseases. Journal of Dental Research, 88(6), pp.490-502*.

@AnnChildersMD tweeted this paper 49 times, her tweets were retweeted 19 times. Twice Childers retweeted retweets of her tweets. Most tweets were the paper title and URL. There were some unique tweets such as these:

*Does it cause tooth decay or gum disease? Avoid* [URL] *#LCHF*

*Is your diet good for your teeth and gums*? [URL]

This began in 2011 and continued through 2016. Although this paper was tweeted 70 times, only 17 accounts were involved.

A third top 10 tweeted paper earned its status thanks to a single-issue campaigner:

*Yu, V. et al. 2016. Electronic cigarettes induce DNA strand breaks and cell death independently of nicotine in cell lines. Oral Oncology, 52, pp.58-65*.

But here there was evidence of broader interest given that 34 accounts tweeted about this paper 39 times, including 30 retweets. The tweeting began a week after the article was posted online, with a tweet and retweet of the title. One month later, on 28 December 2015 the AAAS news service put out a press release about the article that closed with the quote:

"Based on the evidence to date," [the investigator] says, "I believe [e-cigarettes] are no better than smoking regular cigarettes."

The next day retweeting of Clive Bates began. Bates is a British campaigner whose blog—The Counterfactual—is headlined:

*What's the right thing to do? Analytical advocacy—getting beyond the rhetoric of campaigners* (www.clivebates.com)

Bates took issue with the press coverage resulting from the press release in a letter to the Telegraph newspaper, a blog post about the letter, and a five times retweeted tweet:

oddly, the press release didn't mention cig smoke findings. #tooeasilyplayed

Over the next week the title was tweeted/retweeted 5 times, 1 tweet/retweet pair pointed to the full text, 1 tweet took credit for funding the study and 5 tweets were about ecig vapor damaging DNA.

On January 5, 2016 the AAAS posted a clarification/correction to the original press release:

*Contrary to what was stated or implied in much of the news coverage resulting from this news release, the lab experiments did not find that e-cigarette vapor was as harmful to cells as cigarette smoke. In fact, one phase of the experiments, not addressed in the news release, found that cigarette smoke did in fact kill cells at a much faster rate. However, because similar cell-damage mechanisms were observed as the result of both e-vapor and regular cigarette smoke, Dr. Wang-Rodriguez asserts, based on the evidence from the study, that e-cigarettes are not necessarily a healthier alternative to smoking regular cigarettes. As stated in the journal paper and the news release, further research is needed to better understand the actual long-term health effects of e-cigarettes in humans*. https://www.eurekalert.org/pub_releases/2015-12/varc-chs122815.php, *accessed 3.23.2017*

Five months later, Clive Bates posted a critique on the PubMed page of the paper and tweeted about his post. 19 retweets followed.

### Social media managers

Because tweets tend to appear contemporaneously with the paper’s publication and Twitter launched in 2006, only 6% of papers were published earlier. One of these was tweeted 51 times:

*Lamberts, D.M., Wunderlich, R.C. and Caffesse, R.G., 1982. The Effect of Waxed and Unwaxed Dental Floss on Gingival Health: Part I. Plaque Removal and Gingival Response. Journal of Periodontology, 53(6), pp.393-396*.

The first tweet about this paper was at 7:20pm, April 1, 2016:

Does wax make a difference in the effectiveness of dental floss? Check it out: [URL]

Four hours and 24 minutes later, 48 identical tweets from 41 accounts had accumulated, differing only in their shortened URL, meaning they were not retweets.

A similar pattern appeared for another early paper that received 39 tweets, from 39 accounts:

*Gerabek, W.E., 1999. The tooth-worm: historical aspects of a popular medical belief. Clinical Oral Investigations, 3(1), pp.1-6*.

38 of the tweets read:

You've probably heard of bookworms, but what about tooth worms? [URL]

These tweets appeared sporadically and are probably still appearing.

The most likely explanation for these repeated, identical tweets is that the users behind these accounts hired the same company to run their Twitter accounts. That this happened was suggested by the identical recent tweets from accounts that have tweeted the bookworm text. The attraction of hiring a service to produce a well-maintained stream of relevant content is obvious. The service did not need to worry much about overlap in followers because dental practices maintain Twitter accounts mainly to communicate with patients who are geographically localized and so unlikely to follow two accounts from the same account manager. However, the service did need to be fulfilling promises of providing original content, so it was not going to be retweeting. If the going rate for account maintenance is $200 per month, then the bookworm tweeting pattern suggests that someone is grossing at least $93,600 per year from producing social media content for these 39 dentists ($200 per month according to aldersocial.com, April 2017).

### Broader tweeting

A pattern more convincingly demonstrating interest in a paper was seen in the 59 tweets from 41 accounts about:

*Burt, B.A., 2002. Fluoridation and social equity. Journal of Public Health Dentistry, 62(4), pp.195-200*.

The first 14 tweets, on 1/23/15, report not the paper title, but the conclusions, in English and Spanish:

*Study: Fluoridation is "the most effective and practical way" to reduce dental disparities* [URL] *#factsfavorfluoridation*

Sporadic tweeting continues until a slightly mutated version is tweeted 29 times on 4/14/16:

*Researcher: Fluoridation is "the most effective and practical" way to reduce dental disparities* [URL] *#OralHealthEquity*

A 2007 paper tweeted 54 times by 33 accounts started off slowly. The title of the paper:

*Watt, R.G., 2007. From victim blaming to upstream action: tackling the social determinants of oral health inequalities. Community dentistry and oral epidemiology, 35(1), pp.1-11*.

Was tweeted once in 2013 and again in 2015. Then on February 4, 2016, @WelshMountain tweeted the conclusion rather than the title:

*Greater focus is needed on the social determinants that shape a child’s or family’s oral health* [URL] *#Month4Smiles*

Five identical tweets followed that day. Sporadic repeats continued until the hashtag was changed to #OralHealthEquity on April 7, 2016 after which the tweet appeared 35 times in English and Spanish from 22 accounts on April 14, 2016. #OralHealthEquity was the only frequently used hashtag in this dataset that was neither so basic as to be almost a stop word (dentist, dental, teeth) nor used by one account only. It was used by many accounts tweeting about four papers. These two cases suggest people followed this hashtag, as its application to a tweet seemed to result in more retweeting.

Three more papers filled out the top ten most tweeted. One was a paper in the *British Dental Journal* (BDJ) that was tweeted by the BDJ several times, and each time was retweeted. The other two papers were tweeted by 25 accounts tweeting 7 and 2 text variants respectively. The notable characteristic of these last five cases is the low number of text variants tweeted. To calculate the number of variants URLs and account names were removed, and text strings were counted as the same if they differed only by the addition of a punctuation mark, deletion of characters at the end to accommodate the addition of an account name at the start, addition of something like: “study” at the beginning or Spanish translation. Excluding the BDJ paper, four papers were tweeted 176 times, of which 62 were retweets, leaving 114 notionally original tweets. Yet there are just 16 true text variants among these tweets. The tweets were generated not from the paper, but from previous tweets referencing the paper, and yet did not register as retweets. Interpreting more than 16 of these tweets as representing any kind of engagement with the paper seems quite dubious.

### Summary of tweeting of top 10 papers

This examination of the top ten most tweeted papers provided insight into the nature of tweeting about research articles. Table 1 summarizes the discussion, listing the papers examined, a short reminder of the discussion above, the number of citations from Web of Science, number of tweets about the paper, number of accounts tweeting about each paper, number of text variants represented in the tweets and number of tweets that begin with “@”, which possibly represents conversation and is addressed below. These ten cases accounted for 8.4% of the tweets in this dataset. There were three cases of a single account repeat tweeting one paper that concerned harm to humans. That such papers attracted single issue campaigners has been found in other studies [44]. In one case, high levels of tweeting were driven not by interest in the paper per se, but by interest in a dispute over how the paper was portrayed in the press. Including this case, three cases were underpinned by determined single-issue campaigners. BDJ self-tweeting and associated retweeting explained another case. Finally, four cases evinced more convincing signs of broader interest in a journal article. Each of these articles addressed dental public health, and two benefited from association with the popular #oralhealthequity hashtag. Many accounts tweeted, and the papers both are quite well cited in the research literature. Nevertheless, engagement with the paper was not indicated by the tweeting because the tweets were largely identical, even if not retweets. The same phenomenon was more strikingly illustrated in two cases of identical tweeting from a range of accounts, not retweeting. These two papers differed in topic as well. The rest of the top ten papers were about harm to humans or health inequality. The two other cases were about floss and a historical curiosity and so seem plausibly targeted at dental patients. These tweets were likely sent by professional social media account management services.

**Table 1:** Top 10 most tweeted dental papers.

Paper title	Explanation	Publication year	Citations	Tweets	Accounts	Tweet variants	Begins with @
Acetaminophen Old Drug, New Issues.	Single-issue campaigner	2015	9	264	15	71	103
Dietary Carbohydrates and Dental-Systemic Diseases	Single-issue campaigner	2009	36	70	17	30	14
Fluoridation and social equity.	#oralhealthequity	2002	42	59	41	4	0
From victim blaming to upstream action: tackling the social determinants of oral health inequalities	#oralhealthequity	2007	159	54	33	3	0
The Effect of Waxed and Unwaxed Dental Floss on Gingival Health: Part I. Plaque Removal and Gingival Response	Social media manager	1982	17	51	44	2	0
Electronic cigarettes induce DNA strand breaks and cell death independently of nicotine in cell lines	Largely single-issue campaigner	2016	12	39	34	13	2
The tooth-worm: historical aspects of a popular medical belief.	Social media manager	1999	NA	39	39	3	0
Oral Health Literacy among Female Caregivers: Impact on Oral Health Outcomes in Early Childhood	Duplicate tweets	2010	47	35	25	7	0
Why do GDPs fail to recognise oral cancer? The argument for an oral cancer checklist	Retweets of BDJ tweets	2013	6	29	18	13	0
Beyond the DMFT: the human and economic cost of early childhood caries.	Duplicate tweets	2009	103	28	25	2	0

**Fig 1:**
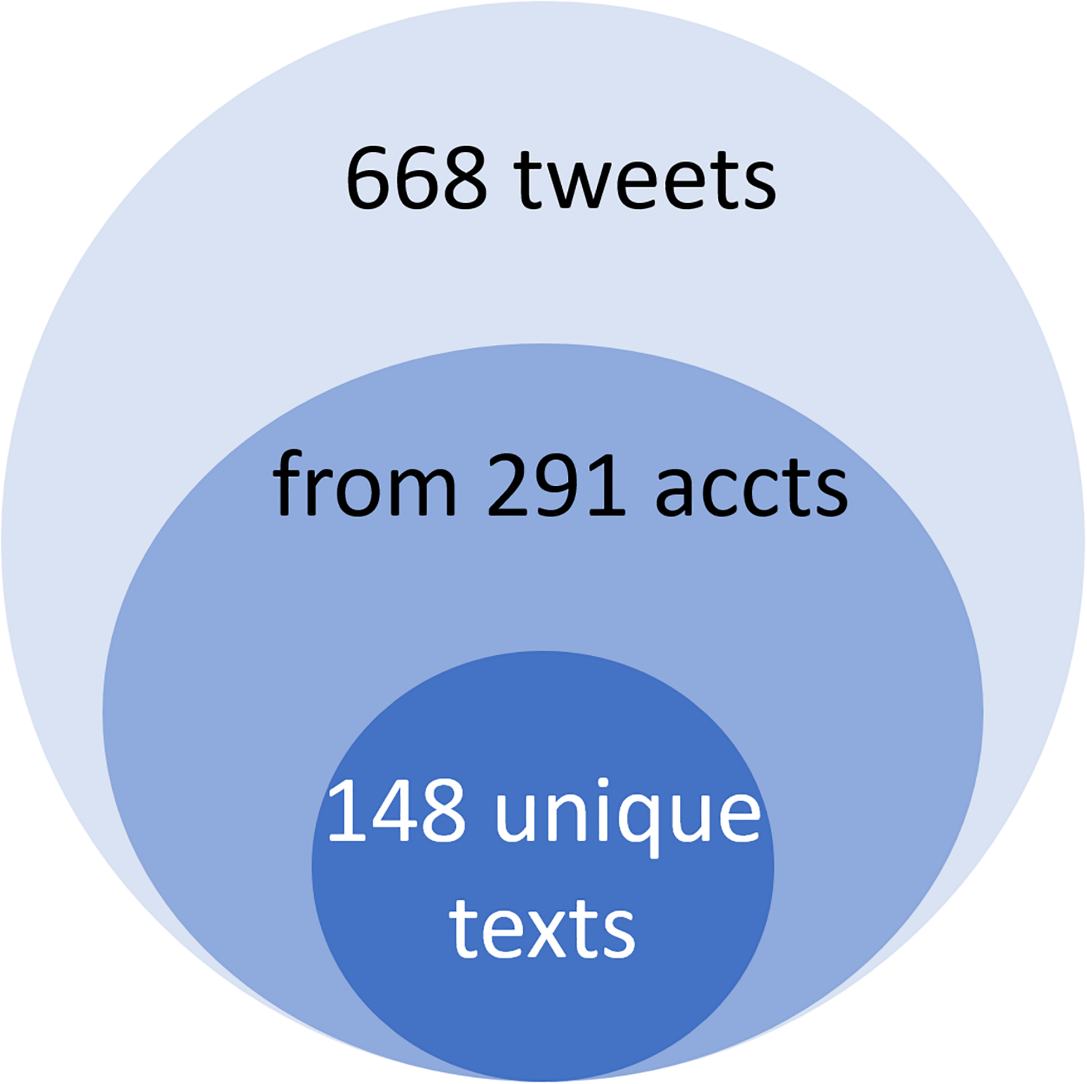
Metrics for the group of 10 most tweeted papers.

Fig 1 illustrates these results. The largest circle represents the number of tweets about these 10 papers– 668. This number decreases to 291 if accounts that tweeted about these 10 papers are counted. Finally, counting unique texts that is non-duplicate tweets, further decreases the total to 148.

### Results—Analysis of twitter accounts

That single accounts played such a large part in the stories of highly tweeted papers points to the importance of characterizing tweeting accounts. 2,202 US based accounts tweeted about dental papers between 2011 and 2016. These accounts posted more than tweets about dental papers, but here only their tweets about dental papers will be characterized. That bots are a substantial presence on Twitter is well known [36,45], suggesting that it is important to determine the presence of bots in this data. The analysis focuses on this, characterizing the tweeting pattern of accounts as mechanical or human.

### What does a tweeting bot look like?

Bots tweeted about dental papers. One bot almost landed a paper in the top 10:

Chou, J.et al., 2014. Bioresorbable zinc hydroxyapatite guided bone regeneration membrane for bone regeneration. *Clinical Oral Implants Research* 27(3), 354–360.

The first red flag was the topic of the paper, which was highly technical in comparison to the cases examined above. Despite its topic, the paper was tweeted 22 times over two days in 2014 by the account @semantic_bot. All the tweets had the form: “True or false?” followed by one word, then two words or phrases in parentheses then a URL for the paper. For example:

True or false? release(#ZnMem, #Pb) [URL] #Collagen

True or false? release(100 thick, buffer solution) [URL] #Collagen

The first sentence of the results section of the abstract was:

*The synthesized **Zn-MEM** (**100** μm **thick**) showed no zinc ions **released** in the phosphate **buffer solution** (PBS) buffer, but zinc was observed under acidic conditions*.

Clearly, this was a Twitter bot grabbing words from the abstract to fill out a formula. This bot tweeted 27 dental papers in 192 tweets over the course of 1 month. The account is gone, a sign that either Twitter deleted it for violation of terms of service or its owner decided to remove it. A second account, @bionavist, tweeted 8 times about dental papers, all were copied @semantic_bot tweets that did not register as retweets. The account racked up 44.2k tweets resending @semantic_bot tweets for about three months in 2014 and has not been deleted.

Another type of bot generated text looks almost, but not quite human (Freitas et al.,2015). For example, this 2014 tweet from @gary_gshafe:

*this is a mouth wash that CAN eliminate bad bacteria LIKE stop cavity's type of good thing? Yes it has bin approve*…

The illogical nature of the tweets is mirrored in the word salad provided as a profile for @gary_gschafe:

Futurist—science & technology- Energy-Transportation- Environment- HEALTH- genetics- seeing Micro-Nano Atomic- Following MR Elon Musk Giving = WORLD

A trio of accounts rounded out the set of bots. @MarkWilliams672, @EmilieThompso15 and @AvaWalker473 appeared in the data with the same one retweet. The last tweet from each account appeared to link to papers but instead linked to a Twitter notice that the link is blocked because it is harmful. The profiles were again word salads:

- Nerd ✦ Playful learning and Jesus ✦ Outdoor enthusiast ✦ Fishing guru
- Psychology and English student ✶ Self-proclaimed foodie ✶ Chocolatier ✶ Happiness Promoter ✶ Christian
- Art photographer | Joyfully at your service | Triathlete | Animal lover | Sailor

Beyond all this, the features that distinguished these accounts and which signaled their bot identity were the large number of accounts they followed and the high friend/follower ratio (i.e. number they follow divided by number who follow them). These features figure prominently among account characteristics found to be useful in identifying fake Twitter accounts, in particular accounts sold as fake followers [46]. @Bionavist, @gary_gschafe, @MarkWilliams672, @EmilieThompso15 and @AvaWalker473 each follow between 1450 and 2000 accounts. Their friend to follower ratio ranged from 6 to 13. On both metrics they scored among the highest in the dataset. It is easy and cheap to buy Twitter followers (buyTwitterfollowersreview.org). If you can sell an account 1,500 time or so (more might arouse Twitter’s suspicions), mounting 1,000 fake accounts could earn you $15,000 at the going rate of $10 per 1,000 followers, more if you dropped customers after a certain amount of time and resold the follow.

These identified bots accounted for 0.14% of accounts and 2.5% of tweets about dental journal articles.

### What does a human tweeting like a bot look like?

Distinguishing humans from bots on Twitter is not easy and is an active area of research [45–47]. It is particularly difficult when examining tweets about journal articles. Truthy, a website that when given an account name rates its likelihood of being a bot, cannot distinguish bots from humans tweeting about papers [36], because one marker of a bot is a high share of tweets containing URLs and this also characterizes tweets about papers.

Chu and colleagues [45] offered several criteria distinguishing humans from bots on twitter: originality, evidence of intelligence and specificity. Most accounts’ tweets about dental papers violated two of Chu et al.’s criteria for identifying a human because they lack original and intelligent content, for example only posting retweets, or repeatedly posting duplicate tweets [45]. For example, 38% of accounts, generating 27% of tweets only retweeted. If humans governed these accounts, their tweeting could easily have been automated, and thus they behaved like a bot. Another 29% of accounts, generating 47% of tweets tweeted only paper titles and URLs. There was no original content in their tweets about dental papers. This result aligns with sentiment analysis of tweets about journal articles that find nothing, leading the authors to conclude that tweeting is ‘unlikely to give insights into the reception of articles by readers’ [48,49]. The title and URL of a paper can be tweeted by clicking on an icon on a paper’s page. Again, this is easily automated and excessive automation was another of Chu et al.’s criteria for being a bot. Humans behaving like bots thus accounted for 67% of accounts and 74% of tweets about dental papers.

Duplicate tweets, that is about the same paper from one account, were not uncommon or confined to a few accounts. Not only does this encompass retweets, but also identical tweets that do not register as retweets. Grabbing the text of a person’s tweet would be an easy way for a bot to avoid gobbledygook. When people engage in this variety of easily automated behavior, they again behave like bots. At minimum, they do not engage with the paper which must cast doubt on interpretations of tweet counts as representing engagement with papers. 216 accounts, or about 10% of the 2,202 examined, tweeted a paper more than once. 52 of these accounts did this for more than one paper.

### What does a human tweeting about papers look like?

A human activity that Twitter might facilitate would be conversations about papers, signaled by tweets beginning with @ and an account name, or tagged by Twitter as replies. Of 343 such tweets, 21 were sent in seven minutes by @JeffNedelman. The tweets read:

New study suggests erythritol may be breakthjrough [sic] approach approach [sic] in oral health. [URL]

Each tweet was prefaced with a different account name, which is perhaps explained by the account profile which states:

*Helping large and small companies communicate nutrition, health & wellness science to top-tier influencers and national media, for more than 25 years*.

Presumably the account names were clients and the tweets were part of the service @JeffNedelman was providing to each. In a somewhat similar gambit, one morning @autismepi decided to tweet 71 times “@[account name] paracetamol research:” followed by three paper URLs. Excluding the 135 tweets that began with @ and were sent by these two accounts and @AnnChilders, 2.5% of tweets here could possibly have been somewhat conversational. This very low percentage indicated that conversation was not driving tweeting about dental papers.

This is not a surprise. The spewing character of much of the tweeting about dental papers bodes ill for potential human readers. Few things so clearly portend information overload than bot imitating, or bot generated text. And information overload means potential human readers retreat, developing filtering and avoidance mechanisms to reduce exposure to a manageable level. Bots of course are unaffected, they can count all night. So the very nature of the tweet flood means nobody is home to enter into conversation.

Our experience working with Twitter data in other domains suggests that even without the volume, Twitter would be ill suited to conversing about papers. In forums or blogs, the concept of a thread applies; a focus is expected. On Twitter, rich conversation invariably signals a veering away from the original tweet with reactions based on how tweeters feel at the time, not exactly stream of consciousness because that implies pure thought, but stream of judgement, attitude or feeling. The e-cigarette case exemplified this in being not about the paper, but rather representing a stream of endorsements for a judgement passed on the press release about a paper.

In search of the human element in tweeting about dental papers, we looked for accounts tweeting more than five times that were definitively human in their tweeting pattern. The leading example was @endofactologist, with 637 tweets, at least 120 referencing papers and 98 tweets about dental papers. Its profile reads: “Endodontic factoids. Get your daily dose”. The account follows 47 and has 312 followers. All the numbers characterizing this account are human scale.

@endofactologist tweeted the conclusions of papers, not the titles. And the topics were quite technical. For example:

- *Endo surgery has better initial success*, *but ReTx offers a more favorable long-term outcome*.
- *There is a dose-response relationship btw cigarette smoking the risk of RCT*.

What was human about this account? First basic facile use of language and syntax removed suspicion of bot generated tweets. Second, the consistent topic interest suggested purpose and intent behind choice of articles creating a larger context of the account’s interest in all things endodontic. This exemplified the ideal of curation Priem and colleagues proposed in the altmetric manifesto [25]. Tweeting the conclusions is possible after someone has read and comprehended a paper, or at least the abstract. This capsule summary approach would offer value to readers in providing more than they could get by glancing at the titles of recent articles in a journal. Overall, 1% of accounts producing 6% of tweets looked like humans actively engaged with the dental literature on Twitter. 31% of the accounts, accounting for 18% of tweets tweeted one or just a couple of links to papers. These accounts may be active dental tweeters, just not consistently interested in journal articles.

### Summary of account tweeting behavior

**Fig 2:**
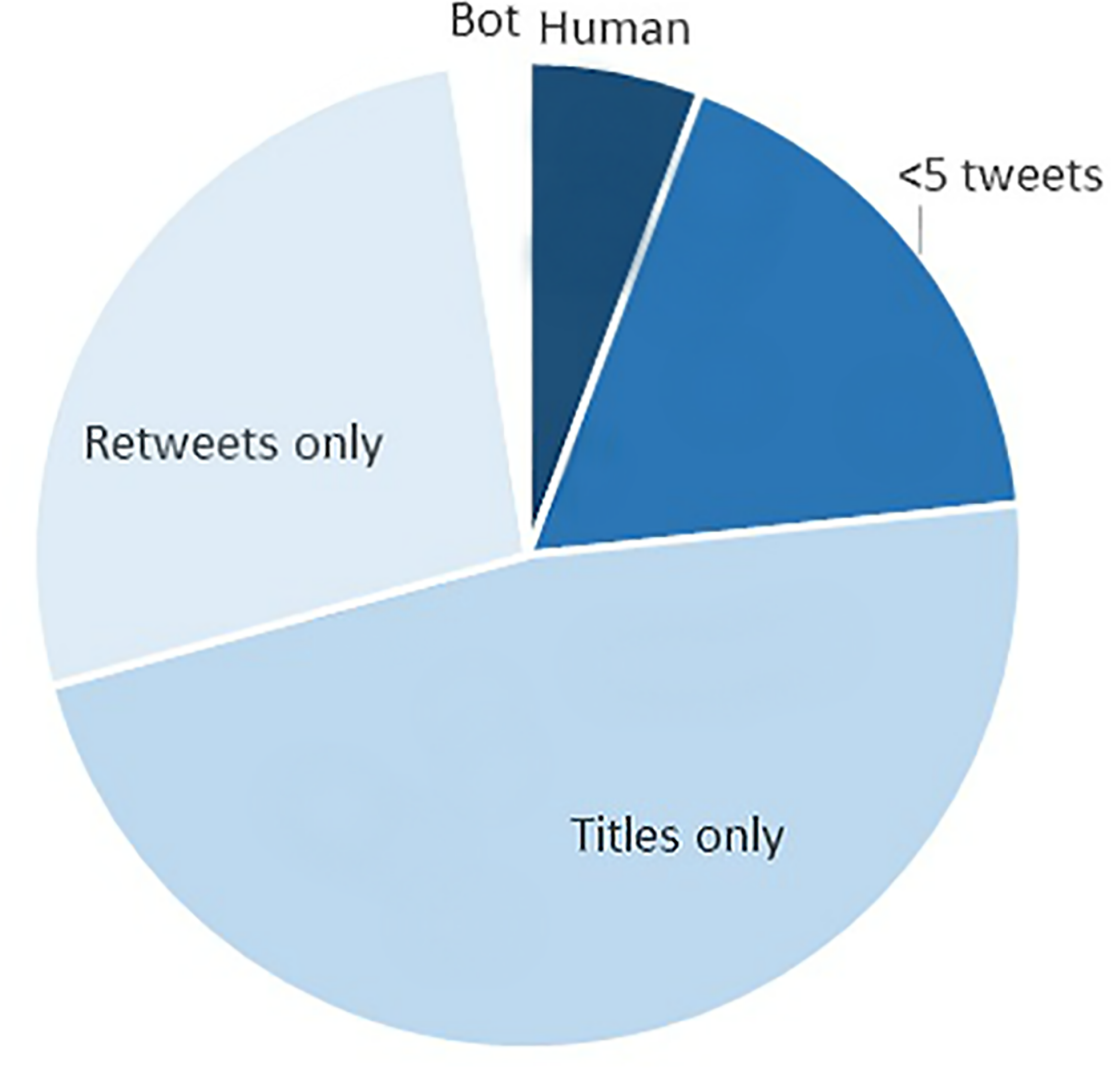
Share of tweets by account type.

Examining the tweeting concerning dental journal articles from 2,202 U.S based accounts provided further insight into the nature of tweeting about research articles. Bots are prevalent on Twitter, and bot accounts tweeted about dental papers. But bots only accounted for 2.5% of tweets. The bulk of tweets about dental papers were sent by accounts seemingly run by people but whose dental journal article tweeting could be easily automated. Thus, they chose to behave like bots in only retweeting or tweeting titles and URLs of papers, easily done by clicking on the Twitter icon on a paper’s webpage. Such tweets lacked the originality that demarcates humans from bots. In contrast, a small percentage of tweets were sent by accounts whose tweets were original, often summarizing the result of a paper. Such accounts have modest metrics—numbers of tweets, followers, friends—as would be accumulated by a person not employed full time to tweet. Fig 2 summarizes these findings.

## Discussion

A multi-year campaign has sought to convince us that counting the number of tweets about papers has value. Yet, reading tweets about dental journal articles suggested the opposite. This analysis found: obsessive single issue tweeting, duplicate tweeting from many accounts presumably under centralized professional management, bots, and much presumably human tweeting duplicative, almost entirely mechanical and devoid of original thought. Advocates of tweeting about the research literature and of altmetrics have extoled an ideal of curating and informing about the literature. Some accounts exemplified this, but they posted less than 10% of tweets about dental papers. Finding these accounts or seeing their influence on Twitter data about dental papers would be like looking for a needle in a haystack.

One response to these findings would be to improve tweet counts by filtering out problematic data. First, the best available algorithms to identify bots and fake follower accounts could be applied to remove such accounts from the data. Analysts of tweeting about papers could develop an open source list of such accounts to be used by everyone so that all analyses meet the same standards of rigor. Second, instead of counting tweets, accounts tweeting about a paper could be counted. This would eliminate the outsized influence of obsessive duplicate tweeting. Altmetric.com does this. The dental dataset would be reduced by 18% from 8,247 tweets, to 6,784 unique account-paper pairs. Third, tweets of the title could be excluded, on the grounds that they lack evidence of engagement with the paper. Finally, if one were concerned to register only evidence of engagement with the paper, unique text variants could be counted. This would eliminate retweets, but as our data revealed, retweets account for only a portion of identical tweets. Cleaning tweets to remove URLs and account names and counting remaining tweet variants for each paper reduces the dental tweet count by 21% to 6,487. Unifying further, across minor text variations such as adding “study” or “researchers” to the beginning of a tweet, changing punctuation or varied number of characters dropped from the end of a tweet when an account name is added to the beginning, would further decrease the unique tweet count.

Rather than using these results to learn how to distill data that aligns with our ideal of what tweeting about papers should be, a second response to these results would be to focus on the part of the data that violates our ideal vision. 74% of tweeting about dental papers was produced by people behaving like bots. If, as one author suggests, “the war for our humanity is upon us” [50], dental tweeting behavior suggests that humanity is losing the war. To understand why people choose to behave like bots requires attending to the affordances of the medium, or the actions Twitter is designed to make as easy as possible. Tweeting is an activity created by a company whose value derives from growth in that activity. The platform is designed to lower barriers to tweeting because the easier it is to tweet, the higher the tweet count and so the higher is the company’s value. Hence the ease of mechanically retweeting and mechanically clicking on a Twitter icon on a page of a journal article. These design features encourage quick, non-human-like action that generates tweets that increase measures of engagement with the platform just as much as a unique, thoughtful, funny piece of text.

In the context of tweeting about journal articles, enrolling people in an algorithmic exercise to maximize tweet counts contrasts with the deeply human activity of advancing knowledge. Even narrowing the focus to the documents involved, journal articles attain their value through a rich association of people working together, expending the resources they have gathered, using equipment and materials, engaging in conceptual and manual labor, writing, and revising. The document implicates people, institutions that support them, materials, equipment, theory and past results on which they build—therefore all can be identified by reading the journal article. Knowledge advances slowly, and endures. It takes time to identify the truly novel advances and incorporate them into further advances [51]. References of course are integral to the narrative in a journal article, functioning to support the argument. By enabling past contributions to be used as resources in building current arguments, references create links across time. The value of references, and their inversion—citations, derives from their entanglement in this nexus.

In contrast, Twitter’s question is: what’s happening? Twitter excels as a medium for the contemporary, immediate and ephemeral. Its forte is relating current news and opinion and enabling asymmetrical connections between individuals and organizations. Its speed favors expressing feelings in the moment rather than thoughtful reflection. Twitter’s immediacy and speed are mismatched to the slow pace of knowledge advance. Its thinned out, 140 character missives contrast with the thick and rich texts of research and scholarship. Twitter’s valuing of volume contrasts with the thoughtfulness of high quality scholarship. The isolation of the tweet and the lack of larger purpose, contrast with the connection indicated by references that serve the larger purpose of the narrative in which they are embedded. And yet, altmetrics was premised on tweets being “faster, broader and more nuanced” [26] and so superior to references when in fact tweets and references are incommensurable because the logic of social media and the institutional logic of the research enterprise differ so radically [52].

## Limitations

We acknowledge the limits of this study. First, one discipline was studied, and things might differ in other areas of science or social science. For example, if a major dental school had been an active tweeter about the research it published, the results would look different, and this could easily happen in another area of research. Second, the analysis of US accounts might not be representative of tweeting behavior in other countries. In particular, the British Dental Journal (BDJ) produced by far the most tweets about dental journal articles and published an article analyzing tweets about dental papers [53], perhaps because it is in the same corporate family as Altmetric.com. Third, tweeting behavior is constantly evolving and our results will date quickly.

## Conclusions

This paper examined 8,000 tweets, from 2,000 US-based accounts referencing 4,000 papers published in dental journals. Examining tweets to the 10 most tweeted papers found that counts would reduce by 56% if accounts tweeting rather than tweets were counted. If unique, non-duplicate tweets were counted, the number would reduce by another 50%, reducing counts to 22% of their original number. Examining tweeting accounts established that bots accounted for 2.4% of tweets, accounts only retweeting for 27%, and accounts just tweeting titles for 47%. In sum, 77% of tweets were mechanical in nature. 23% of tweets showed a more human, creative character though most such accounts tweeted about dental papers only a few times. 6% of accounts were human in character and sustained their tweeting about dental papers.

The results suggest that analysts who would use counts of tweets either to evaluate research or as a source of data should exercise caution. At minimum, bots and fake followers should be identified (and preferably eliminated) and unique accounts tweeting rather than tweets should be counted. Better would be to identify and discount accounts with identical tweets. Even better would be to count true unique text variants. Of course, doing this would reduce Twitter data to a shadow of its current self, which would not be in the interests of the advocates currently pushing for its use. However, it would be in the interests of analysts to identify the tweets and accounts that are truly informative, relevant and indicative of reception and discussion of research.

Any evaluative regime risks establishing incentives that do not align with the desired result, in this case research excellence. Research evaluation by tweet count would promise particular dangers of this kind, above and beyond the standard litany of problems associated with metrics regimes applied to research. Evaluation by tweet count would present scientists with incentives to work on the kind of oddball topics that attract obsessive single issue campaigners or are useful to social media account managers. Focusing on tweet counts might lead to impatience with the stately, all-too-human, pace at which knowledge advances. Finally, tweet counts are all-too-easily inflated, enticing people to allocate limited time to serving the bot agenda, a service that will always be better provided by bots themselves.

What is more, the dangers extend to librarians who recommend readings to students and professionals based on such metrics in the belief that they are truly providing an added value. Almetric.com’s famous doughnut displaying the altmetric score of papers, appearing on ever more journal websites, is largely driven by Twitter counts. Its capacity to focus attention in a time when scientific literature increases exponentially, might harm those tempted to confound well-conducted and highly rigorous research with inflated numbers of tweets the origin and meaning of which is, to say the least, obscure. Should altmetrics gain in legitimacy and influence as superfluous evaluative devices, the danger to the research enterprise would grow.

Simplistic and naïve use of social media data risks damaging the scientific enterprise, misleading both authors and consumers of scientific literature. All users of Twitter data need to be mindful of its limits and consider whether it is fit for the purposes for which they propose to use it.

## References

[1] Twitter in retweet. The Economist [Internet]. 2016 Sep 17 [cited 2017 May 5]; http://www.economist.com/news/business/21707258-it-too-late-social-media-firm-become-giant-people-once-expected-twitter

[2] Eysenbach G. Medicine 2.0: social networking, collaboration, participation, apomediation, and openness. J Med Internet Res. 2008;10(3):e22 doi: 10.2196/jmir.1030 18725354 10.2196/jmir.1030 PMC2626430

[3] Hawn C. Take two aspirin and tweet me in the morning: how Twitter, Facebook, and other social media are reshaping health care. Health Aff (Millwood). 2009;28(2):361–368. 19275991 10.1377/hlthaff.28.2.361

[4] McGinnigle E, McAloon CJ, Warriner DR, Francis R, Chohan BC. Analysis of cardiologists’ use of twitter. Eur Heart J. 2016 8 1;37:1264–1264.

[5] Alpert JM, Womble FE. Just What the Doctor Tweeted: Physicians’ Challenges and Rewards of Using Twitter. Health Commun. 2016 7;31(7):824–32. doi: 10.1080/10410236.2015.1007551 26644165 10.1080/10410236.2015.1007551

[6] Roupret M, Misrai V. Exponential use of social media in medicine: Example of the interest of Twitter (c) in urology. Prog Urol. 2015 1;25(1):11–7. 25454777 10.1016/j.purol.2014.10.009

[7] Nason GJ, O’Kelly F, Kelly ME, Phelan N, Manecksha RP, Lawrentschuk N, et al The emerging use of Twitter by urological journals. Bju Int. 2015 3;115(3):486–90. doi: 10.1111/bju.12840 24925047 10.1111/bju.12840

[8] Forgie SE, Duff JP, Ross S. Twelve tips for using Twitter as a learning tool in medical education. Med Teach. 2013;35(1):8–14. doi: 10.3109/0142159X.2012.746448 23259608 10.3109/0142159X.2012.746448

[9] Maclean F, Jones D, Carin-Levy G, Hunter H. Understanding Twitter. Br J Occup Ther. 2013 6;76(6):295–8.

[10] Grande D, Gollust SE, Pany M, Seymour J, Goss A, Kilaru A, et al Translating Research For Health Policy: Researchers’ Perceptions And Use Of Social Media. Health Aff (Millwood). 2014 7 1;33(7):1278–85. 24907363 10.1377/hlthaff.2014.0300

[11] Schnitzler K, Davies N, Ross F, Harris R. Using Twitter (TM) to drive research impact: A discussion of strategies, opportunities and challenges. Int J Nurs Stud. 2016 7;59:15–26. doi: 10.1016/j.ijnurstu.2016.02.004 27222446 10.1016/j.ijnurstu.2016.02.004

[12] Cardona-Grau D. Join the discussion on Twitter! J Pediatr Urol. 2016 12;12(6):334–334. doi: 10.1016/j.jpurol.2016.11.003 27889219 10.1016/j.jpurol.2016.11.003

[13] Fuller MY, Allen TC. Let’s Have a Tweetup The Case for Using Twitter Professionally. Arch Pathol Lab Med. 2016 9;140(9):956–7. doi: 10.5858/arpa.2016-0172-SA 27195434 10.5858/arpa.2016-0172-SA

[14] Thompson MA, Majhail NS, Wood WA, Perales M-A, Chaboissier M. Social Media and the Practicing Hematologist: Twitter 101 for the Busy Healthcare Provider. Curr Hematol Malig Rep. 2015 12 1;10(4):405–12. doi: 10.1007/s11899-015-0286-x 26449718 10.1007/s11899-015-0286-x PMC4679678

[15] Haustein S, Peters I, Bar-Ilan J, Priem J, Shema H, Terliesner J. Coverage and adoption of altmetrics sources in the bibliometric community. Scientometrics. 2014 1 4;101(2):1–19.

[16] Letierce J, Passant A, Breslin J, Decker S. Understanding how Twitter is used to spread scientific messages. In Raleigh, NC: US; 2010 [cited 2014 Feb 19]. http://journal.webscience.org/314/

[17] Costas R, Zahedi Z, Wouters P. Do ‘altmetrics’ correlate with citations? Extensive comparison of altmetric indicators with citations from a multidisciplinary perspective. J Assoc Inf Sci Technol. 2015;66(10):2003–2019.

[18] Robinson-García N, Torres-Salinas D, Zahedi Z, Costas R. New data, new possibilities: exploring the insides of Altmetric. com. El Prof Inf. 2014;23(4):359–366.

[19] Sugimoto CR, Work S, Larivière V, Haustein S. Scholarly use of social media and altmetrics: a review of the literature. ArXiv Prepr ArXiv160808112 [Internet]. 2016 [cited 2017 Jan 26]; https://arxiv.org/abs/1608.08112

[20] Thelwall M, Haustein S, Larivière V, Sugimoto CR. Do Altmetrics Work? Twitter and Ten Other Social Web Services. PLoS ONE. 2013 5 28;8(5):e64841 doi: 10.1371/journal.pone.0064841 23724101 10.1371/journal.pone.0064841 PMC3665624

[21] Haustein S, Costas R, Larivière V. Characterizing Social Media Metrics of Scholarly Papers: The Effect of Document Properties and Collaboration Patterns. PLOS ONE. 2015 3 17;10(3):e0120495 doi: 10.1371/journal.pone.0120495 25780916 10.1371/journal.pone.0120495 PMC4363625

[22] Haustein S, Tsou A, Minik V, Brinson D, Hayes E, Costas R. Identifying Twitter user communities in the context of altmetrics. In Bucharest; 2016.

[23] Eysenbach G. Can Tweets Predict Citations? Metrics of Social Impact Based on Twitter and Correlation with Traditional Metrics of Scientific Impact. J Med Internet Res. 2011;13(4):e123 doi: 10.2196/jmir.2012 22173204 10.2196/jmir.2012 PMC3278109

[24] Piwowar H, Priem J. RLM: Investigating metrics at the researcher level. In Bucharest; 2016.

[25] Priem J, Taraborelli P, Groth C, Neylon C. altmetrics: a manifesto—altmetrics.org [Internet]. 2010 [cited 2014 Feb 13]. http://altmetrics.org/manifesto/

[26] Priem J, Costello KL. How and why scholars cite on Twitter. Proc Am Soc Inf Sci Technol. 2010;47(1):1–4.

[27] Choo EK, Ranney ML, Chan TM, Trueger NS, Walsh AE, Tegtmeyer K, et al Twitter as a tool for communication and knowledge exchange in academic medicine: A guide for skeptics and novices. Med Teach. 2015 5;37(5):411–6. doi: 10.3109/0142159X.2014.993371 25523012 10.3109/0142159X.2014.993371

[28] Adie E. Taking the Alternative Mainstream. Prof Inf. 2014 8;23(4):349–51.

[29] Konkiel S, Madjarevic N, Rees A. Altmetrics for librarians: 100+ tips, tricks, and examples [Internet]. Altmetric LLP; 2016. http://dx.doi.org/10.6084/m9.figshare.3749838

[30] Kramer B. #osc2017 What % of researchers use (and librarians recommend) #altmetrics tools/platforms cf. traditional metrics? 101innovations.wordpress.com [Internet]. 2017. https://Twitter.com/i/web/status/844539651083919361

[31] Wilsdon J, al. The Metric Tide: Report of the Independent Review of the Role of Metrics in Research Assessment and Management. 2015. Report No.: 10.13140/RG.2.1.4929.1363.

[32] Bornmann L. Alternative metrics in scientometrics: a meta-analysis of research into three altmetrics. Scientometrics. 2015 6 1;103(3):1123–44.

[33] Haustein S, Peters I, Sugimoto CR, Thelwall M, Larivière V. Tweeting biomedicine: An analysis of tweets and citations in the biomedical literature. J Assoc Inf Sci Technol. 2014;65(4):656–69.

[34] Ke Q, Ahn Y-Y, Sugimoto CR. A systematic identification and analysis of scientists on Twitter. PLOS ONE. 2017 abr;12(4):e0175368 doi: 10.1371/journal.pone.0175368 28399145 10.1371/journal.pone.0175368 PMC5388341

[35] Zahedi Z, Costas R, Wouters P. How well developed are altmetrics? A cross-disciplinary analysis of the presence of ‘alternative metrics’ in scientific publications. Scientometrics. 2014;101(2):1491–1513.

[36] Haustein S, Bowman TD, Holmberg K, Tsou A, Sugimoto CR, Larivière V. Tweets as impact indicators: Examining the implications of automated ‘bot’ accounts on Twitter. J Assoc Inf Sci Technol. 2016;67(1):232–238.

[37] Torres-Salinas D, Cabezas-Clavijo Á, Jiménez-Contreras E. Altmetrics: New Indicators for Scientific Communication in Web 2.0. Comunicar [Internet]. 2013 [cited 2016 Feb 4];21(41). http://arxiv.org/abs/1306.6595

[38] Haustein S. Grand challenges in altmetrics: heterogeneity, data quality and dependencies. Scientometrics. 2016;1–11.26798160

[39] Wouters P, Costas R. Users, Narcissism and Control—Tracking the Impact of Scholarly Publications in the 21 st Century. [cited 2014 Feb 13]; http://2012.sticonference.org/Proceedings/vol2/Wouters_Users_847.pdf

[40] Tufekci Z. Big questions for social media big data: Representativeness, validity and other methodological pitfalls. ArXiv Prepr ArXiv14037400 [Internet]. 2014 [cited 2017 May 5]; https://arxiv.org/abs/1403.7400

[41] Zimmer Michael, Proferes Nicholas John. A topology of Twitter research: disciplines, methods, and ethics. Aslib J Inf Manag. 2014 5 19;66(3):250–61.

[42] Robinson-Garcia N, Costas R, Isett K, Melkers J, Hicks D. Underlying data to the study: The unbearable emptiness of tweeting—about journal articles [Internet]. 2017. https://figshare.com/articles/Underlying_data_to_the_study_The_unbearable_emptiness_of_tweeting_-_about_journal_articles/5195122/2 10.1371/journal.pone.0183551 PMC5570264 28837664

[43] Altmetric.com. How does Altmetric track Twitter? [Internet]. 2016 [cited 2017 Jul 13]. https://help.altmetric.com/support/solutions/articles/6000157183-how-does-altmetric-track-twitter-

[44] Vainio J, Holmberg K. Highly tweeted science articles: who tweets them? An analysis of Twitter user profile descriptions. Scientometrics. 2017 4 1;1–22.

[45] Chu Z, Gianvecchio S, Wang H, Jajodia S. Detecting automation of twitter accounts: Are you a human, bot, or cyborg? IEEE Trans Dependable Secure Comput. 2012;9(6):811–824.

[46] Cresci S, Di Pietro R, Petrocchi M, Spognardi A, Tesconi M. Fame for sale: Efficient detection of fake Twitter followers. Decis Support Syst. 2015 12;80:56–71.

[47] Freitas C, Benevenuto F, Ghosh S, Veloso A. Reverse engineering socialbot infiltration strategies in twitter. In: Proceedings of the 2015 IEEE/ACM International Conference on Advances in Social Networks Analysis and Mining 2015 [Internet]. ACM; 2015 [cited 2017 May 5]. p. 25–32. http://dl.acm.org/citation.cfm?id=2809292

[48] Thelwall M, Tsou A, Weingart S, Holmberg K, Haustein S. Tweeting Links to Academic Articles. Cybermetrics Int J Scientometr Informetr Bibliometr. 2013;(17):1–8.

[49] Friedrich N, Bowman TD, Stock WG, Haustein S. Adapting sentiment analysis for tweets linking to scientific papers. In: Salah AA, Tonta Y, Salah A a. A, Sugimoto C, Al U, editors. Proceedings of Issi 2015 Istanbul: 15th International Society of Scientometrics and Informetrics Conference. Leuven: Int Soc Scientometrics & Informetrics-Issi; 2015. p. 107–8.

[50] Philp D. A $500 House in Deroit: Rebuilding an abandoned home and an American city. Scribner; 2017.

[51] Wang X, Fang Z, Guo X. Tracking the digital footprints to scholarly articles from social media. Scientometrics. 2016 7 29;1–12.26798160

[52] Van Dijck J, Poell T. Understanding social media logic. 2013 [cited 2017 May 5]; https://papers.ssrn.com/sol3/papers.cfm?abstract_id=2309065

[53] Kolahi J, Khazaei S. Altmetric: Top 50 dental articles in 2014. Br Dent J. 2016 6 10;220(11):569–74. doi: 10.1038/sj.bdj.2016.411 27283563 10.1038/sj.bdj.2016.411

